# Temporal Dynamics in Neuroimmunomodulation and Aging: Moving Beyond Snapshot Biology

**DOI:** 10.1159/nim/adxag007

**Published:** 2026-09-16

**Authors:** Alexander K.H. Weiss

**Affiliations:** Institute for Biomedical Aging Research, University of Innsbruck, Innsbruck, Austria

**Keywords:** Neuroimmunomodulation, Aging, Temporal dynamics, Mitochondria, Resilience

Neuroimmunomodulation is a science of regulation over time. Neural, endocrine, immune, and metabolic processes do not merely occupy high or low levels. They oscillate, respond to perturbation, compensate across systems, and recover. Aging research addresses the same temporal problem: not only whether a biological measure is elevated or reduced, but whether flexibility, coordination, and resilience change across the life course. Yet literature linking stress, mental health, immunity, mitochondria, and aging often interprets biomarker observations obtained at a single sampling occasion, or cross-sectional differences between participants, as indicators of stable vulnerability, biological embedding, or accelerated aging [1–6]. Here, a single biological sample means a biospecimen or biomarker measurement obtained from an individual at one sampling occasion, without repeated observation of that biological readout over time.

This mismatch is more than a technical limitation. It is a conceptual blind spot. Psychological constructs such as chronic stress, depression, trauma exposure, and resilience are defined over weeks, years, or cumulative histories. Many biological readouts used to index them are short-lived and context sensitive. Cytokines vary with circadian phase, recent infection, sleep, physical activity, medication, and acute challenge. Cortisol is pulsatile and governed by feedback. Mitochondrial respiration changes with immune activation, substrate availability, temperature, pH, cell composition, and energetic demand [7, 8]. A single sample therefore measures a biological state under distinct conditions. By itself, it does not establish a biological trait.

The language of the field often moves too quickly across levels of inference. A higher inflammatory marker becomes chronic inflammation. A lower respiratory readout becomes mitochondrial dysfunction. A telomere or methylation measure becomes accelerated aging [1, 2, 7]. These interpretations may ultimately be correct, but observation alone rarely proves them. A trait-level claim requires evidence that the state is persistent or recurrent, predicts a trajectory, or reflects a reproducible alteration in regulation. A claim of dysfunction requires a further step: evidence that the pattern impairs performance, adaptation, or recovery rather than simply marking a different physiological state.

This distinction matters because the same cross-sectional difference can arise through different mechanisms. An elevated inflammatory signal may reflect persistent dysregulation, a current infection, altered leukocyte composition, recent sleep loss, or an appropriate response to an unresolved demand. A reduced mitochondrial readout in peripheral blood mononuclear cells may reflect intrinsic capacity, a shift in lymphocyte and monocyte proportions, substrate conditions, or transient immune activation. These alternatives are not interchangeable because each assigns the observed value to a different biological process. They therefore imply different causal models, different prognoses, different follow-up measurements, and potentially different interventions. For example, persistence would be tested by repeated measurements, a cell-composition explanation by cell-resolved or composition-adjusted analyses, and context dependence by standardized or experimentally manipulated sampling conditions.

Aging makes the temporal problem especially visible. Aging is not defined by one biological level at one time point. It is defined by trajectories in repair, rhythmicity, stress responsivity, immune coordination, metabolic flexibility, and recovery. Cross-sectional comparisons can show that groups differ under specified sampling conditions. They cannot, without additional assumptions, show that one group is aging faster. A group can appear biologically older because of a stable between-person difference, a reversible current state, selective survival, cohort effects, or a true difference in longitudinal rate.

The distinction between biological age and rate of aging is therefore essential. Biomarkers such as DNA methylation age and telomere length can be informative summaries of accumulated biology [1, 2], but a single measurement does not directly reveal velocity. Even repeated measurements require attention to assay precision, regression to the mean, and the timescale on which meaningful change is expected. In studies of stress and mental health, the relevant question is often not simply whether a marker differs, but whether the organism has lost the capacity to return to baseline, maintain coordination across systems, or adapt to repeated challenge [3–6].

Time of day, sleep, infection, physical activity, medication, hormonal state, food intake, and recent stress are frequently treated as nuisance variables. Standardization and covariate adjustment are often necessary, but a regulatory system cannot be understood by treating all context as noise. Context may be the route through which psychological experience becomes biology. It can determine whether the same biomarker value is adaptive, compensatory, exhausted, or pathological.

The practical implication is not that every study must measure everything. It is that sampling conditions should be interpreted rather than merely controlled. A biomarker result should be described as conditional on the observed context, and the context most likely to alter its meaning should be reported transparently. When context is deliberately manipulated, as in a stress, exercise, immune, or metabolic challenge, it becomes an experimental probe of regulation. Reactivity, peak response, coordination, and recovery can then be studied directly instead of inferred from a resting value.

Mitochondria illustrate the broader problem particularly well. They integrate endocrine, immune, metabolic, and redox signals, which makes them attractive candidates for connecting psychological stress with systemic aging [7, 8]. At the same time, mitochondrial measurements are multilevel readouts. They may reflect intrinsic organelle capacity, cellular activation, substrate conditions, tissue heterogeneity, recent signaling history, or the proportions of cell types in the sample. In peripheral blood, a group difference may arise from altered respiration per cell, from different numbers of metabolically distinct immune cells, or from a transient inflammatory environment.

Calling each difference mitochondrial dysfunction collapses levels of explanation that should remain separate. A more precise account specifies what was measured, in which cells or tissue, under which assay conditions, and whether the result persisted, predicted function, or changed after challenge. The same discipline applies to inflammatory markers, endocrine measures, epigenetic clocks, and telomeres. The closer the conclusion is to damage or disease, the more temporal and functional evidence it requires.

Temporal inference is not achieved simply by collecting more samples. The sampling schedule must match the dynamics of the biological system and the claim being tested. Dense sampling over hours can characterize pulses and circadian structure. For example, serial cortisol measurements before and after a standardized psychosocial stressor can distinguish baseline level, peak reactivity, and recovery, whereas one morning cortisol value cannot. Repeated cytokine measurements across a day can separate a sustained elevation from a transient value occurring near a circadian or behavioral transition. Repeated sampling across days can estimate short-term variability and state dependence. Measurements across months or years can address persistence and trajectories, for example, whether an inflammatory marker or epigenetic-age estimate remains stable, normalizes, or changes at a different rate. Challenge-and-recovery designs can reveal reserve capacity and regulatory flexibility. An immune, exercise, or metabolic challenge can be followed by serial sampling to determine response amplitude, time to peak, coordination across systems, and time to return toward baseline. These designs answer different questions and should not be treated as substitutes.

For many neuroimmuno-modulatory questions, the most informative outcomes may be dynamic rather than static: amplitude of response, latency to peak, coupling between systems, speed of recovery, variability around a set point, and the degree to which repeated perturbations produce habituation or sensitization. A high resting value may be less consequential than a delayed recovery. A low value may reflect efficient downregulation rather than failure. Two individuals with the same baseline may therefore differ substantially in reserve capacity and resilience: after the same standardized challenge, one may mount an appropriately sized response and return rapidly toward baseline, whereas the other may show a blunted or exaggerated response, poor coordination between systems, or delayed recovery. The baseline value alone cannot reveal these differences.

A useful design hierarchy follows from this logic. Cross-sectional studies can establish conditional group differences. Repeated resting measures can estimate stability and recurrence. Longitudinal studies can test direction and rate of change. Perturbation studies can examine reactivity and recovery. Designs combining repeated baselines with standardized challenges offer the clearest route to distinguishing a persistent trait from a context-bound state. When these designs are not feasible, the interpretation should remain at the level supported by the data.

Model organisms such as *Caenorhabditis elegans* and mice can contribute because they permit experimentally controlled observation of mitochondrial function, behavior, stress resistance, immune responses, and lifespan within an organismal framework. Their strongest contribution to this perspective is temporal. They allow investigators to manipulate the timing, duration, and recurrence of stressors; separate developmental from adult effects; and test whether altered states recover, persist, or predict later decline. Work linking mitochondrial enzymes, behavior, and longevity in *C. elegans* illustrates the value of placing molecular readouts within an organismal trajectory [9]. A complementary design is provided by Norden and colleagues, who compared adult and aged mice after a standardized peripheral immune challenge and followed the post-challenge response to show that aging was associated not simply with a different inflammatory level, but with impaired resolution of microglial activation [10]. The comparison is instructive: Ref. [9] links molecular perturbation to organismal phenotypes and longevity across aging, whereas the challenge-and-recovery design in Ref. [10] makes the temporal property of failed resolution experimentally visible.

Such models do not replace human biomarker studies, and their temporal advantages do not erase translational limits. Rather, they can sharpen human questions. A model can identify which dynamic property is mechanistically informative, which sampling interval captures it, and which recovery pattern predicts later dysfunction. Human studies can then test whether the corresponding dynamic signature is observable in clinically relevant contexts.

The central methodological question is which design makes the temporal property of interest observable. Repeated resting measurements are appropriate when the goal is to estimate stability, recurrence, within-person variability, or circadian organization. Longitudinal cohort designs are required when the claim concerns direction or rate of change across months or years. Standardized perturbation designs are particularly informative for neuroimmunomodulation because they convert context from a nuisance into a probe of regulation. Psychosocial stress, exercise, vaccination or other immune stimulation, and metabolic challenges can be paired with serial neural, endocrine, immune, or metabolic measurements to quantify reactivity and recovery. Ambulatory and wearable measurements can complement biosampling by locating biological observations within sleep-wake cycles, physical activity, autonomic state, and daily stress exposure.

Analytically, these designs call for outcomes that preserve time rather than collapse it. Depending on the question, useful quantities include within-person variability, slope, area under the response curve, peak amplitude, latency to peak, recovery time, phase and amplitude of rhythms, cross-system coupling, and habituation or sensitization across repeated challenges. Multilevel and longitudinal models can separate within-person change from between-person differences, while functional or time-series approaches can compare the shapes of response trajectories. No single method is universally preferable. The design and analysis should be chosen from the biological timescale and the inferential claim. When temporal designs are not feasible, static measurements remain useful, but conclusions should be limited to the state and sampling conditions actually observed.

The point is not to abandon peripheral biomarkers or cross-sectional studies. It is to interpret them according to the temporal properties of the systems they measure. Neuroimmunomodulation cannot be understood through static measurements alone. The field needs more explicit attention to state, context, reactivity, coordination, and recovery, and greater use of repeated, longitudinal, and perturbation-based designs when the claim concerns persistence, dysfunction, resilience, or aging rate. Progress may depend less on discovering new biomarkers than on asking temporally precise questions of the markers already in use.

## Conflict of Interest Statement

The author has no conflicts of interest to declare.

## Funding Sources

This study was not supported by any sponsor or funder.

## Author Contributions

A.K.H.W. conceived the perspective, drafted the manuscript, revised it critically for intellectual content, and approved the final manuscript.
